# Glymphatic System Alterations in Lung Cancer Patients with Chemotherapy-Related Cognitive Impairment

**DOI:** 10.2174/0115734056415041251203091854

**Published:** 2026-03-16

**Authors:** Lanyue Hu, Miaomiao Chang, Shuo Li, Yujie Zhang, Jia You, Qian Li, Yu-Chen Chen, Yuan Feng, Xindao Yin

**Affiliations:** 1 Department of Radiology, Nanjing First Hospital, Nanjing Medical University, Nanjing, China; 2 Department of Neurology, Nanjing Yuhua Hospital, Yuhua Branch of Nanjing First Hospital, Nanjing, China; 3 Department of Respiratory Medicine, Nanjing First Hospital, Nanjing Medical University, Nanjing, China

**Keywords:** Non-small cell lung cancer, Chemotherapy, Cognitive impairment, DTI-ALPS, Choroid plexus volume, Neurocognitive changes, NSCLC survivors

## Abstract

**Background:**

Chemotherapy-related cognitive impairment (CRCI) is a common adverse effect among lung cancer patients undergoing chemotherapy. Glymphatic system dysfunction has been implicated in the pathogenesis of cognitive deficits. This study aimed to investigate the impact of chemotherapy on the glymphatic system in patients with non-small cell lung cancer (NSCLC) and its association with cognitive outcomes.

**Methods:**

A total of 102 NSCLC patients (53 patients without chemotherapy (CT(-)), 49 patients with chemotherapy (CT(+))) and 56 healthy controls (HCs) were enrolled. Their glymphatic system function was evaluated using the diffusion tensor imaging analysis along the perivascular space (DTI-ALPS) index and choroid plexus (CP) volume, derived from DTI and three-dimensional T1-weighted magnetic resonance imaging (3D-T1 MRI), and examined for group differences and associations with neuropsychological outcomes.

**Results:**

The MoCA scores of NSCLC survivors decreased significantly after chemotherapy. There were significant differences in the DTI-ALPS index and CP volume among the three groups ( *P <* 0.05, one-way ANOVA). Post hoc analyses (Bonferroni-corrected) demonstrated that the CT(+) group exhibited significantly reduced DTI-ALPS indices and enlarged CP volumes compared to both the CT(–) group and the HCs group ( *P <* 0.05). DTI-ALPS indexes were positively correlated with MoCA scores (r = 0.448, *P* = 0.001), and CP volumes were negatively correlated with cognitive performance (r= -0.435, *P* = 0.002). Mediation analysis indicated that DTI-ALPS indexes partially mediated the relationship between CP volumes and MoCA scores (mediated effect = 32.79%).

**Conclusion:**

This research provides new insights into the pathological mechanism of CRCI in patients with NSCLC. These results suggest that glymphatic system-derived metrics (DTI-ALPS indices and CP volume) might act as potential biomarkers for assessing CRCI and tracking neurocognitive changes in NSCLC patients receiving chemotherapy.

## 1 INTRODUCTION

Chemotherapy-related cognitive impairment (CRCI) is one ofthemostcommonadverseresponsestochemotherapy, which can lead to extensive decline of cognitive functions in patients [ 1-3]. Given the critical importance of chemotherapy in treating non-small cell lung cancer (NSCLC) patients, CRCI significantly impacts the quality of life and functional capacity of NSCLC survivors [4-7]. Neuroimaging research has uncovered widespread changes in the brain after chemotherapy, particularly in the frontoparietal and limbic areas. However, the mechanisms linking chemotherapy to ongoing cognitive impairments remain poorly understood [8-13]. Fortunately, recent advances in glymphatic studies provide new insights into the processes behind cognitive deterioration linked to chemotherapy.

The glymphatic system is a waste removal system recently discovered in the central nervous system (CNS). The main functions of this system include nutrient transport, waste removal, and immune regulation [ 14 ]. Studies have found that dysfunction of the glymphatic system is related to many neurodegenerative diseases [ 15-17]. The glymphatic system maintains the steady state of the CNS through perivascular fluid exchange, which is interrelated with the regulation of neuroinflammation [18-23]. Chemotherapeutic agents may disrupt this balance in two ways: (1) breaking the blood-brain barrier (BBB), allowing drugs and cytokines into the brain parenchyma; (2) microglia are activated by proinflammatory cytokines, such as TNF-α and IL-6, resulting in neuroinflammation [24-29]. These processes may destroy the transport, clearance, and immune functions of the glymphatic system, leading to neuron damage and cognitive decline. Although the evidence shows that glymphatic dysfunction in breast cancer is related to CRCI [30], this relationship has not been explored in NSCLC due to different chemotherapy regimens and neurotoxicity characteristics. Thus, further investigation into the relationship between CRCI and glymphatic function in NSCLC is required.

Recently, some non-invasive MRI indices can be used to indirectly evaluate the function of the lymphoid system. Diffusion tensor image analysis along the perivascular space (DTI-ALPS) is an MRI technique that does not require the injection of a contrast agent. It can quantify the level of directional water diffusion in the brain. The DTI-ALPS index obtained from this method can be used as a new neuroimaging biomarker to directly assess and quantify the functional state of the glymphatic system. At present, DTI-ALPS has been proposed to be a reliable and effective indicator for evaluating the activity of the human glymphatic system [ 31 ]. As for CRCI, only one study revealed impaired glymphatic system function in breast cancer patients using this method [ 30 ]. However, no previous study investigated the relationship between glymphatic system function and CRCI in lung cancer patients. In addition, the choroid plexus (CP) is also one of the important indicators of glymphatic system function [ 32, 33]. It has been reported that chemotherapy drugs, such as methotrexate, can impair the secretory function of the CP, thereby causing the impaired clearance of CSF [23]. CP volume enlargement has been widely reported in neurodegenerative and neuroinflammatory diseases [34, 35], but few studies have focused on its relationship with chemotherapy-related cognitive impairment.

In the present study, we used the DTI-ALPS index and CP volume to investigate abnormal changes in glymphatic activity in NSCLC patients before and after chemotherapy. We further examined associations between these glymphatic indices and cognitive performances in NSCLC patients after chemotherapy. We hypothesized that: (a) compared with health controls, patients after chemotherapy show significant alterations in DTI-ALPS index and CP volume; (b) DTI-ALPS index and CP volume are correlated with cognitive outcomes; (c) DTI-ALPS index plays a partial mediating role in the process of CP volume affecting cognitive function. Our findings may provide a theoretical basis for preventing and treating CRCI in NSCLC by improving the function of the glymphatic system.

## 2 MATERIALS AND METHODS

### 2.1 Participants

This cross-sectional study was conducted from May 2023 to August 2024. Fifty-five chemotherapy-naïve (CT(-)) and 55 chemotherapy-treated (CT(+)) lung cancer patients were enrolled from the Department of Respiratory Medicine at Nanjing First Hospital. Additionally, 60 age-matched healthy controls (HCs) were recruited through online advertisements and nearby local community networks. All participants were right-handed and had completed primary education or higher. For the CT(-) group, MRI was performed after NSCLC diagnosis but before any chemotherapy. For the CT(+) group, MRI was conducted 3–6 months after completing chemotherapy. For the initial enrolled participants, further screening was conducted based on the inclusion and exclusion criteria. The inclusion criteria were as follows: (1) initial diagnosis of NSCLC (stages II–III) confirmed by surgical, bronchoscopic, or percutaneous biopsy; (2) for chemotherapy patients, completion of a platinum-based regimen over 3–6 months; and (3) absence of brain metastases at diagnosis or during follow-up. The exclusion criteria were as follows: (1) history of prophylactic cranial irradiation or metastatic brain tumor; (2) history of stroke, traumatic brain injury, epilepsy, Alzheimer’s disease, Parkinson’s disease, or other major neurological or psychiatric disorders; and (3) significant medical comorbidities, such as anemia, severe cardiac disease, thyroid dysfunction, or hepatic/renal impairment.

### 2.2 Neuropsychological Testing

All subjects were assessed with a neuropsychological scale to evaluate their cognitive status. General cognitive function was evaluated using the Mini-Mental State Examination (MMSE) [ 36 ]. MMSE has a total score of 30, and a total score of ≤26 is considered cognitive decline. The Montreal Cognitive Assessment (MoCA) was used to evaluate patients' cognitive functions, such as visuospatial/executive function, attention, language, abstraction, memory (immediate and delayed recall), and spatial orientation [ 37 ]. The total score of MoCA is 30 points, and a score < 26 points is considered cognitive decline. The test was conducted in a quiet environment, and all the subjects maintained a conscious state of relaxation.

### 2.3 MRI Acquisition

All subjects completed MRI data collection within 1-2 days of completing the cognitive test. MRI data were collected using the same 8-channel 3.0T magnetic resonance scanner (Ingenia, Philips Medical Systems, Netherlands). Earplugs were provided to reduce scanner noise, while tight and comfortable foam pads were used to prevent head movement. The subjects were required to keep their eyes closed, remain calm, and awake during the scanning. We performed conventional MRI sequences on each subject, including thick-slice T1-weighted imaging, T2-weighted imaging, and T2 fluid-attenuated inversion recovery (FLAIR) imaging, to exclude any brain organic lesions or brain metastases.

Diffusion tensor images (DTI) were obtained using a single-shot echo planar imaging (EPI) sequence. The scan parameters were as follows: TR = 6600 ms; TE = 95 ms; FA = 90°; 70 slices; thickness = 2 mm; no gap; b-values = 0 and 1000 s/mm^2^; 30 diffusion directions; matrix = 128×128; FOV = 256×256 mm. 3D-T1 images were acquired using a 3D turbo fast echo (3D-TFE) T1WI sequence. The scan parameters were as follows: TR = 8.1 ms; TE = 3.7 ms; FA = 8°; 172 slices; thickness = 1 mm; no gap; matrix = 256×256; FOV = 256×256 mm.

### 2.4 Data Processing

#### 2.4.1 DTI-ALPS Index Calculation

DTI-ALPS indices were derived from diffusion-weighted images using the DTIFIT tool in FSL (FMRIB Software Library, University of Oxford, https://fsl.fmrib.ox.ac.uk/fsl/fslwiki/FSL) [31, 38]. The specific processing process includes the following steps: (1) DICOM-to-NIFTI conversion using MRIcron; (2) preprocessing in DSI Studio (format conversion, skull stripping, eddy current correction, tensor calculation); (3) placement of 5-mm spherical regions of interest (ROIs) over bilateral projection, association, and subcortical fibers on color-coded FA maps using FSLeyes; subsequently, we extracted the diffusivities of the three projection tracts along the x, y, and z axes at the ROIs on the bilateral fibers. Furthermore, (4) the DTI-ALPS index was calculated using the following formula (Eq. **1**):

**Table:** 

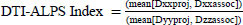	(1)

Dxxproj: diffusivity along the x- axis in the projection fiber. Dxxassoc: diffusivity along the x- axis in the association fiber. Dyyproj: diffusivity along the y- axis in the projection fiber. Dzzassoc: diffusivity along the z- axis in the association fiber.

ROIs were independently marked by two radiologists with 10 years of working experience to ensure the reliability among observers. The intra-group correlation coefficient (ICC) was 0.88.  Fig. (**1**)  shows the calculation pipeline of DTI-ALPS.

#### 2.4.2 Choroid Plexus Volume Measurement

U-Shaped Neural Network (U-NET) architecture (https://github.com/Center-of-maging-Biomarker-Development/chp_seg) based on a full convolution neural network model was adopted to automatically segment the choroid plexus volume [39]. The architecture was verified in the adults, and the potential errors inherent in the current manual segmentation technology were minimized. The detailed processing steps are as follows: (1) non-linear registration of 3D-T1 images to the International Consortium for Brain Mapping-Montreal Neurological Institute (ICBM-MNI) 152 -T1-weighted template using Advanced Normalization Tools (ANTs) [40]; (2) The image, after being processed by the 3D U-NET fully convolutional neural network, underwent an inverse transformation back to its native imaging space. This step produced a mask of the CP. Each generated mask was then superimposed on the original image and reviewed by an expert; (3) The volume of the CP was calculated utilizing SimpleITK (version 2.1.1.1) within Python (3.7.0); (4) Computational Anatomy Toolbox (CAT12, Jena University Hospital, Departments of Psychiatry and Neurology, Germany, https://github.com/ChristianGaser/cat12) in Statistical Parametric Mapping software package (SPM12, Functional Imaging Laboratory, Wellcome Department of Cognitive Neurology, UCL Queen Square Institute of Neurology, UK, http://www.fil.ion.ucl.ac.uk/spm) was utilized to segment T1 images and obtain various volume measurements. This included determining the total intracranial volume (TIV), gray matter volume (GMV), white matter volume (WMV), and cerebrospinal fluid volume (CSFV); (5) Values were normalized as a ratio relative to TIV multiplied by 1000, in order to mitigate the effects of individual differences in brain size; subsequent analyses were carried out by utilizing this standardized ratio. In a pool of 30 randomly selected individuals, a radiologist, unaware of the clinical information and other imaging details, independently performed manual segmentation of the CP in the lateral ventricle using ITK-SNAP software, version 3.8.0 (available at http://www.itksnap.org). The manual segmentation showed a significant Spearman correlation with the automatically segmented volume measurements, as evidenced by a correlation coefficient of 0.85 (*P <* 0.05). Fig. (**2**) shows CP volume across groups.

### 2.5 Statistical Analysis

Data were analyzed using SPSS 26.0 (SPSS, Inc., Chicago, IL, USA) and GraphPad Prism 10.2.0 (GraphPad Software, Boston, Massachusetts, USA).

Categorical variables were compared using chi-square tests. Continuous variables were compared *via* one-way ANOVA with Bonferroni post-hoc tests (*p <* 0.05). All data are presented as mean±standard deviation (SD). Two-tailed Spearman correlations assessed relationships between glymphatic metrics (DTI-ALPS index and CP volume) and MoCA scores among patients undergoing platinum-based chemotherapy, with age, gender, education, baseline cognition, chemotherapy regimen, and tumor stage as covariates (FDR-corrected, *p <* 0.05).

In addition, based on the significant correlations identified in the aforementioned analyses, we employed the PROCESS macro for SPSS (version 4.3) to conduct a mediation analysis. This was carried out to examine the interactions among the ALPS index, CP volume, and cognitive function. Significance was set at *p <* 0.05 or 95% CI excluding zero.

## 3 RESULTS

### 3.1 Demographic and Clinical Features

During the data collection and image processing stages, a number of participants were excluded for specific reasons: (1) 2 patients in the CT(-) group were excluded due to excessive head motion (>3mm or 3°) during MRI acquisition. (2) In the CT(+) group, 6 patients were excluded for the following reasons: 2 due to excessive head motion (>3mm or 3°), 1 due to claustrophobia that prevented successful completion of MRI scanning, and 3 due to inability to tolerate the prolonged duration of MR imaging protocols. (3) Among HCs, 4 indivi-duals were excluded: 1 due to claustrophobia and 3 due to failure to complete the required cognitive assessment scales. Consequently, the final cohort included in the analysis comprised 53 CT(-) patients, 49 CT(+) patients, and 56 HCs. Groups did not differ significantly in gender, age, education, or MMSE scores (*p* >0.05). The prevailing histological diagnoses across both patient groups, regardless of their chemotherapy history, comprised squamous cell carcinoma and adenocar-cinoma. In the CT(+) group, 29 received cisplatin-based and 20 received carboplatin-based chemotherapy over 4–6 cycles. Mean time post-chemotherapy was 157.4 ± 65.2 days. We compared baseline cognitive function, chemotherapy regimens, and tumor staging within the CT(+) group and found no sta-tistically significant differences (*p* > 0.05). MoCA scores were significantly lower in the CT(+) group compared to HCs (*p <* 0.001). Demographic and clinical data are summarized in Table **1**.

### 3.2 Group Differences in MRI Indices of Glymphatic Function

**Fig. (3):**
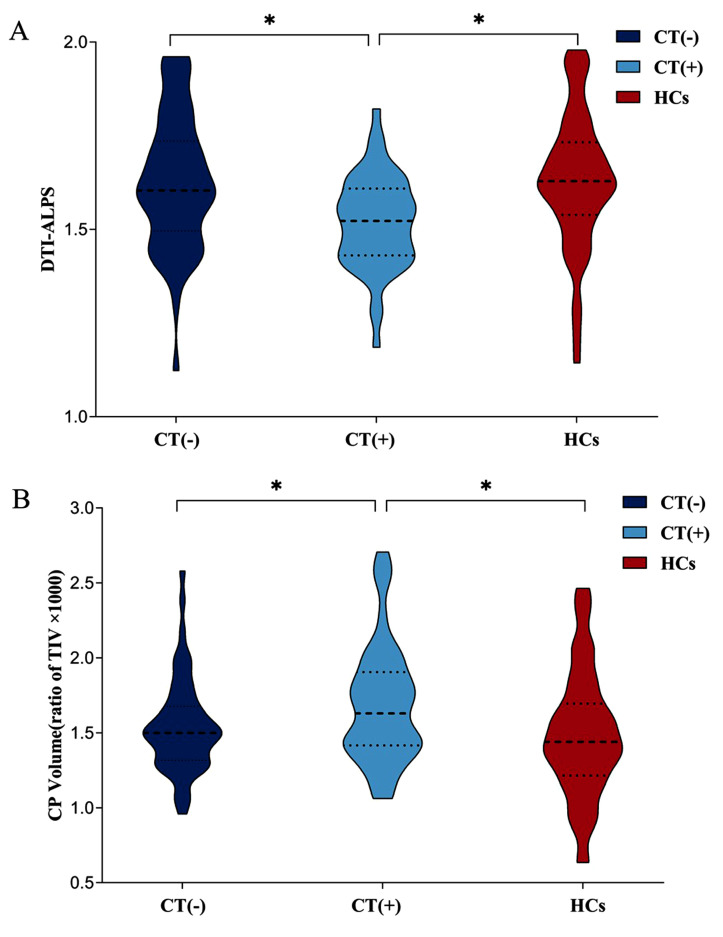
Differences between the glymphatic system functions of CT(-), CT(+), and HCs. (**A**) Comparison of the DTI-ALPS index in three groups. The Fig. (shows that the DTI-ALPS index of CT(+) is significantly lower than that of CT(-) and HCs. (**B**) Comparison of CP volume in three groups. The Fig. (shows that the CP Volume of CT(+) is significantly larger than that of CT(-) and HCs. DTI-ALPS: Diffusion Tensor Image Analysis Along the Perivascular Space. CP volume: Choroid Plexus Volume. CT(-): Non-Chemotherapy Group; CT(+): Chemotherapy Group; HCs: Healthy Control Group.

Group differences in MRI indices of glymphatic function among the three groups are shown in Table **2**. Significant group differences were found in ALPS index (F=6.729, p=0.002) and CP volume (F=4.049, p=0.019). Post-hoc analysis indicated that the DTI-ALPS indexes were significantly lower in the CT(+) group compared to the CT(-) group and HCs (*p*<0.05), while there was no significant difference between the CT(-) group and HCs (Fig. **3A**). Besides, Post-hoc tests showed that the CT(+) group had significantly larger CP volumes than both CT(-) and HCs (*p <* 0.05). CP volume was higher in CT(-) than in HCs, but not significantly (Fig. **3B**).

### 3.3 Correlation Analysis

**Fig. (4):**
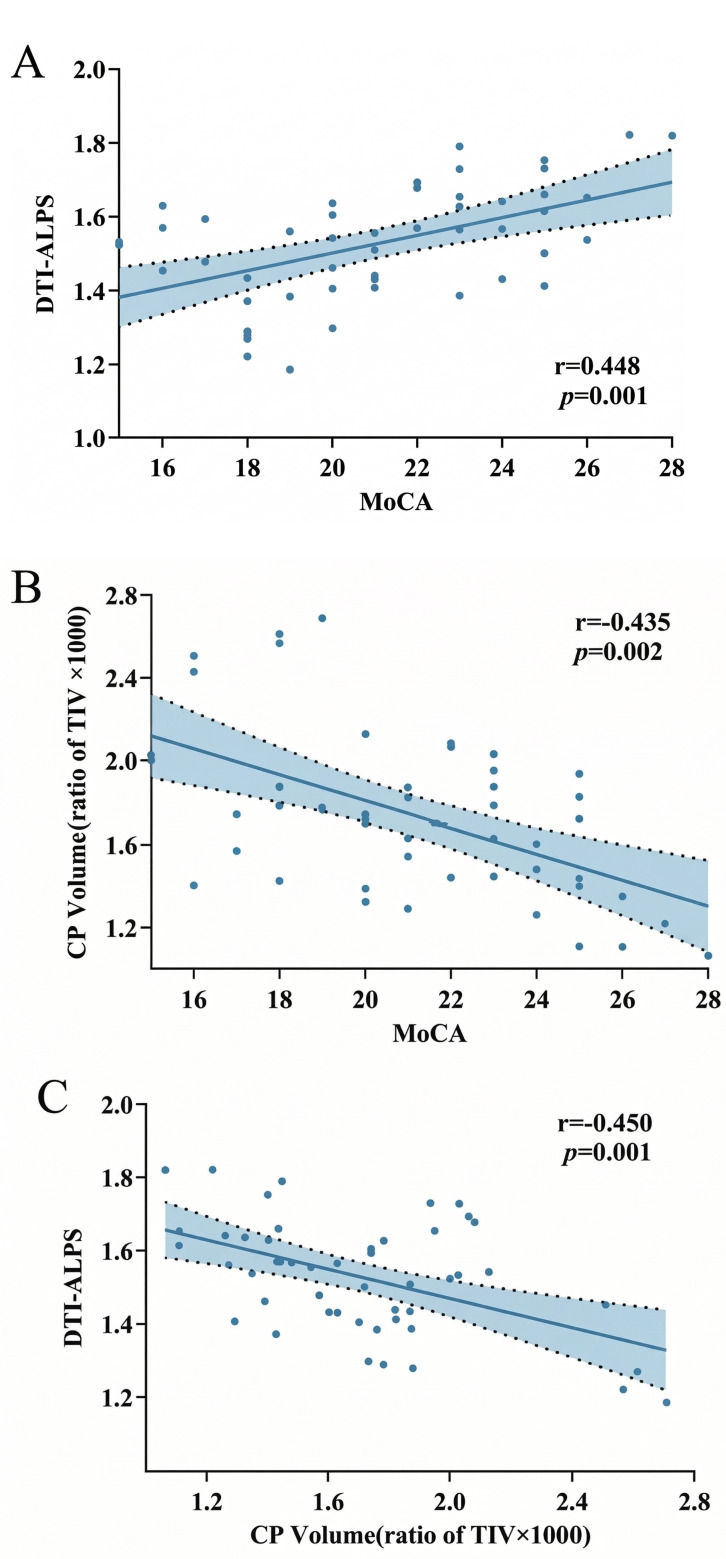
Correlation between glymphatic system function and cognitive function in the CT(+) group. (**A**) Positive correlations between diffusion tensor image analysis along the perivascular space (DTI-ALPS) index and MoCA scores (r = 0.448, p = 0.001). (**B**) Negative correlations between CP volume (ratio TIV ×1000) and MoCA scores (r = 0.435, p = 0.002). (**C**) Negative correlations between CP volume (ratio TIV ×1000) and DTI-ALPS index (r =-0.450, p=0.001). CT(-): Non-Chemotherapy Group; CT(+): Chemotherapy Group; HCs: Healthy Control Group. DTI-ALPS: Diffusion Tensor Imaging Along the Perivascular Space. CP volume: Choroid Plexus Volume. MoCA: Montreal Cognitive Assessment.

In the CT(+) group, DTI-ALPS index correlated positively with MoCA scores (r=0.448, p=0.001), while CP volume correlated negatively with MoCA scores (r=-0.435, p=0.002). CP volume also correlated negatively with DTI-ALPS index (r=-0.450, p=0.001). All correlations survived FDR correction (*p <* 0.05) (Fig. **4**).

### 3.4 Mediation Analysis

Figure (**5**) shows the simple mediation analysis of glymphatic function and cognitive factors in patients after chemotherapy. DTI-ALPS index partially mediated the association between CP volume and MoCA scores after adjusting for age and education (indirect effect standardized β=-0.1425, *p <* 0.05, mediated effect=32.79%).

## 4 DISCUSSION

This study is the first to explore the contribution of glymphatic activity in the mechanism of cognitive impairment in patients with non-small cell lung cancer after chemotherapy. We used DTI data to calculate the DTI-ALPS index and structural MRI to evaluate CP volume. Our results showed that the DTI-ALPS index decreased significantly, and the CP volume increased significantly in NSCLC patients after chemotherapy. These changes have a steady correlation with cognitive function scores. Moreover, the DTI-ALPS index partially mediated the effect of CP enlargement on cognitive performance. These glymphatic metrics may be used as biomarkers and targets for monitoring the progress and prevention of CRCI in NSCLC patients.

The DTI-ALPS index can be used to measure the diffusion intensity of water molecules around blood vessels and is an important parameter for evaluating glymphatic system function [ 31 ]. The lower DTI-ALPS index means that the diffusion of perivascular water diffusivity is worse, which indicates that the glymphatic circulation function is impaired [ 41 ]. Previous studies reported significant correlations between DTI-ALPS index and cognitive ability scores among patients with Alzheimer's disease, Parkinson's disease, and traumatic brain injury [ 17, 42-45]. A recent study revealed a significant decline in the DTI-ALPS index and a significant increase in cerebrospinal fluid volume in patients with brain tumors [46]. Our results are consistent with the above results, and the DTI-ALPS index of lung cancer patients decreased significantly after chemotherapy. We speculate that chemotherapeutic drugs may trigger an inflammatory reaction [47] and interfere with the function of astrocytes [26], leading to a decrease in perivascular diffusion, thus damaging the function of the glymphatic system. However, this finding is contrary to a study based on breast cancer patients after chemotherapy, which demonstrated that the DTI-ALPS index increased slightly compared with the pre-chemotherapy and healthy control groups [30]. This difference may be related to differences in cancer types and chemotherapy schemes, and further research is needed through a large-scale prospective cohort study.

It is well known that CSF is mainly produced by the choroid plexus and partially flows back to the brain through the glymphatic circulation, which extends along the vascular space around the cerebral arteries [ 48 ]. Therefore, there is a potential possibility that the choroid plexus and the glymphatic system can communicate with each other through the circulation of CSF. Furthermore, the choroid plexus not only synthesizes CSF but also integrates inflammatory signals from the central nervous system and circulating immune cells [ 49 ]. Changes in CP volume were considered a potential biomarker of glymphatic system dysfunction caused by neuroinflammation [ 50 ]. The mechanism underlying the increase in CP volume associated with chemotherapy is unclear. However, others have reported that chemotherapy drugs have harmful effects on the oxidative metabolism of non-neoplastic choroid plexus epithelial cells and CSF [ 23 ]. Previous animal studies have demonstrated that chemotherapy drugs can cause oxidative phosphorylation in the choroid plexus and damage the antioxidant defense of cerebrospinal fluid to the brain parenchyma, thus increasing drug toxicity in the brain *via* cerebrospinal fluid and causing neurological structural and functional damage [ 51, 52]. Furthermore, it was found that the volume of choroid plexus (CP) in the CT(-) group was larger than that in the healthy group. It may be due to damage to the lymphatic system from lung cancer. Further research is needed on the specific mechanism behind this. It is reasonably to conclude that an increase in CP volume may mean the enhanced cerebrospinal fluid production potential by increasing functional units.

This phenomenon may be a compensatory mechanism for the functional damage of the lymphoid system.

Notably, our analysis also found a significant negative correlation between the decrease in DTI-ALPS index and the increase in choroid plexus (CP) volume with a decrease in cognitive function in the chemotherapy group of NSCLC survivors, which further indicated that the larger CP volume was related to the decrease in the clearance efficiency of cerebrospinal fluid. Additionally, the intermediary analysis demonstrated that the decrease in the DTI-ALPS index partially mediated the effect of increased CP on cognitive impairment. These findings indicate that the increased CP volume may lead to CRCI by reducing the efficiency of the CSF clearance system. Previous studies reported that the content of oxidative stress markers in the hippocampus of rats increases after chemotherapy [ 53 ], which may be one of the mechanisms of cognitive dysfunction caused by chemotherapy [ 23 ]. Under the influence of cancer and chemotherapeutic drugs, CP may be involved in the changes in cognitive function through cerebrospinal fluid secretion and inflammatory regulation [ 50 ]. Given the above evidence, we speculate that chemotherapeutic drugs may alter glymphatic function indices, which may eventually lead to cognitive dysfunction. Although this is an interesting finding, further mechanistic research is needed to determine how chemotherapeutic drugs cause cognitive impairment in NSCLC survivors by affecting glymphatic system indices.

This study has several limitations. Firstly, this study is a cross-sectional survey. Although healthy controls and NSCLC patients were included before and after chemotherapy, there was a lack of longitudinal follow-up of patients, which increased the influence of confounding factors. Therefore, a larger, longitudinal sample-size study (including before chemotherapy, three months after chemotherapy, and more than one time point in a year) is needed to verify the results of this study. Secondly, numerous studies have reported that neuroinflammation may play a role in the pathophysiological mechanism of CRCI, and it also plays a key role in glymphatic dysfunction. However, no inflammatory markers were collected in this study. In the future, we plan to integrate the inflammatory markers TNF-α, IL-1β, and IL-6 to evaluate the role of inflammatory factors in the occurrence of CRCI in NSCLC patients. Thirdly, regarding the time interval after chemotherapy (average 157.4±65.2 days), although it was the best time to observe the changes in the brain related to chemotherapy, to choose a time window of 3-6 months according to the literature [54], we acknowledge the potential influence of the time interval range. Our future analysis will divide the time interval into subgroups of 3-4 months and 5-6 months to further explore its influence on the results. Finally, the index of lymphoid function is the whole brain index. Previous studies have found that chemotherapy has different effects on the structure and function of different brain regions (*e.g.*, the hippocampus and the frontotemporal cortex). Therefore, future research can combine structural and functional MRI data to better understand the neural mechanisms underlying the effects of chemotherapy drugs on cognitive function.

## CONCLUSION

In summary, this research provides new insights into the pathological mechanisms underlying CRCI and glymphatic system dysfunction in NSCLC patients, focusing particularly on the key roles of the decline in DTI-ALPS index and the increase in CP volume. Our results show that these specific glymphatic indexes may serve as potential biomarkers for early detection and treatment intervention in CRCI in NSCLC patients.

## Figures and Tables

**Fig. (1):**
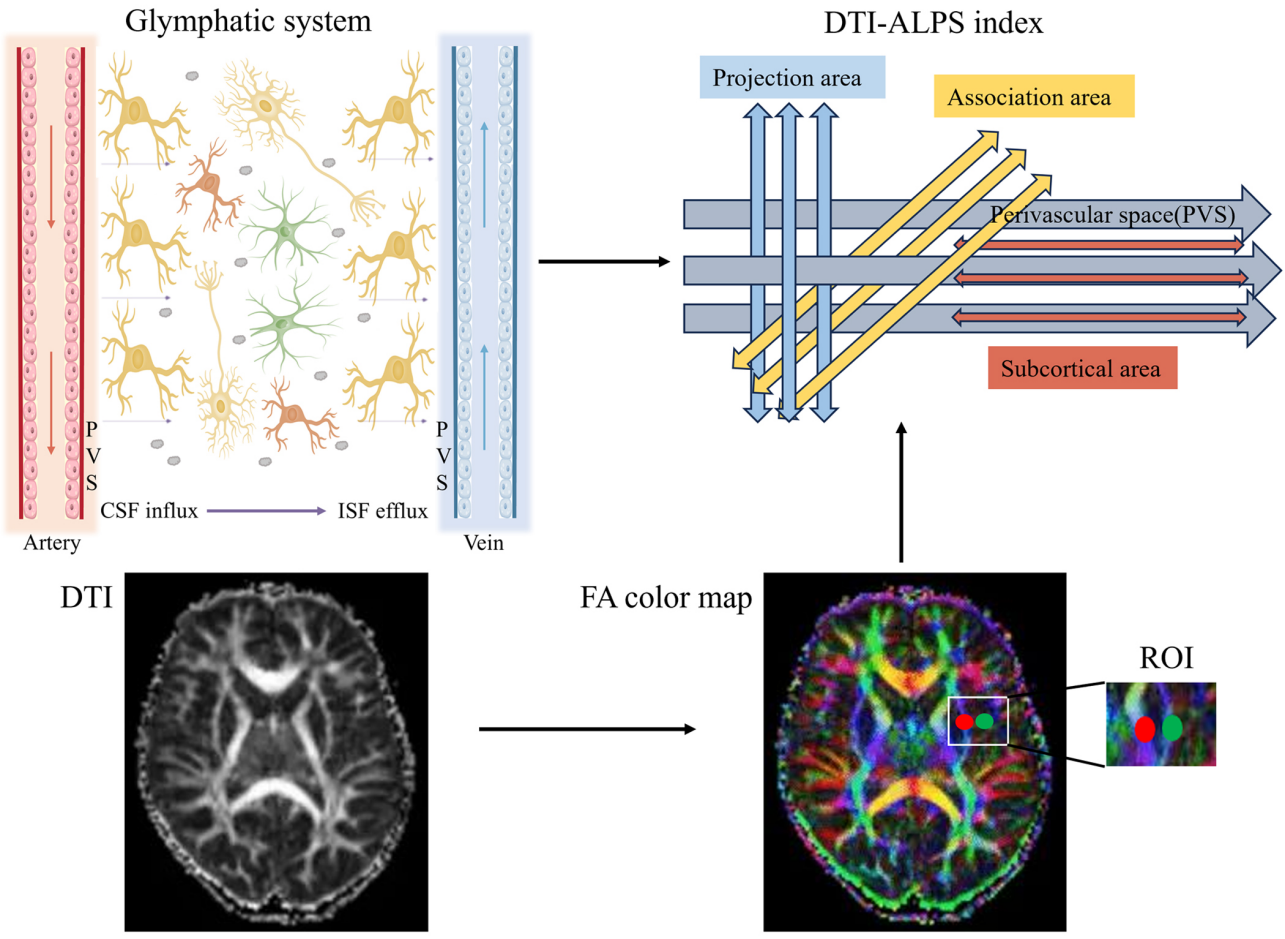
Flowchart for DTI-ALPS index calculation method. Firstly, we present a schematic diagram of the glymphatic system, briefly mapping the course of CSF inflow, the interstitial system, and ISF flow. Subsequently, the analysis process from the initial DWI image to the calculation of the DTI-ALPS index is briefly shown: (1) Preprocessing of raw DTI data; (2) ROI delineation: Anatomical localization using the MNI152 standard brain atlas, with manual adjustment to ensure alignment with individual anatomical features; (3) ALPS index computation: Extracted the diffusivities of the three projection tracts along the x, y, and z axes at the ROIs on the bilateral fibers, and the DTI-ALPS index was calculated using the following formula: DTI-ALPS Index =”(mean[Dxxproj, Dxxassoc])” /”(mean[Dyyproj, Dzzassoc])” DTI-ALPS: diffusion tensor imaging along the perivascular space. DWI: diffusion tensor imaging. CSF: cerebrospinal fluid. ISF: interstitial fluid.

**Fig. (2):**
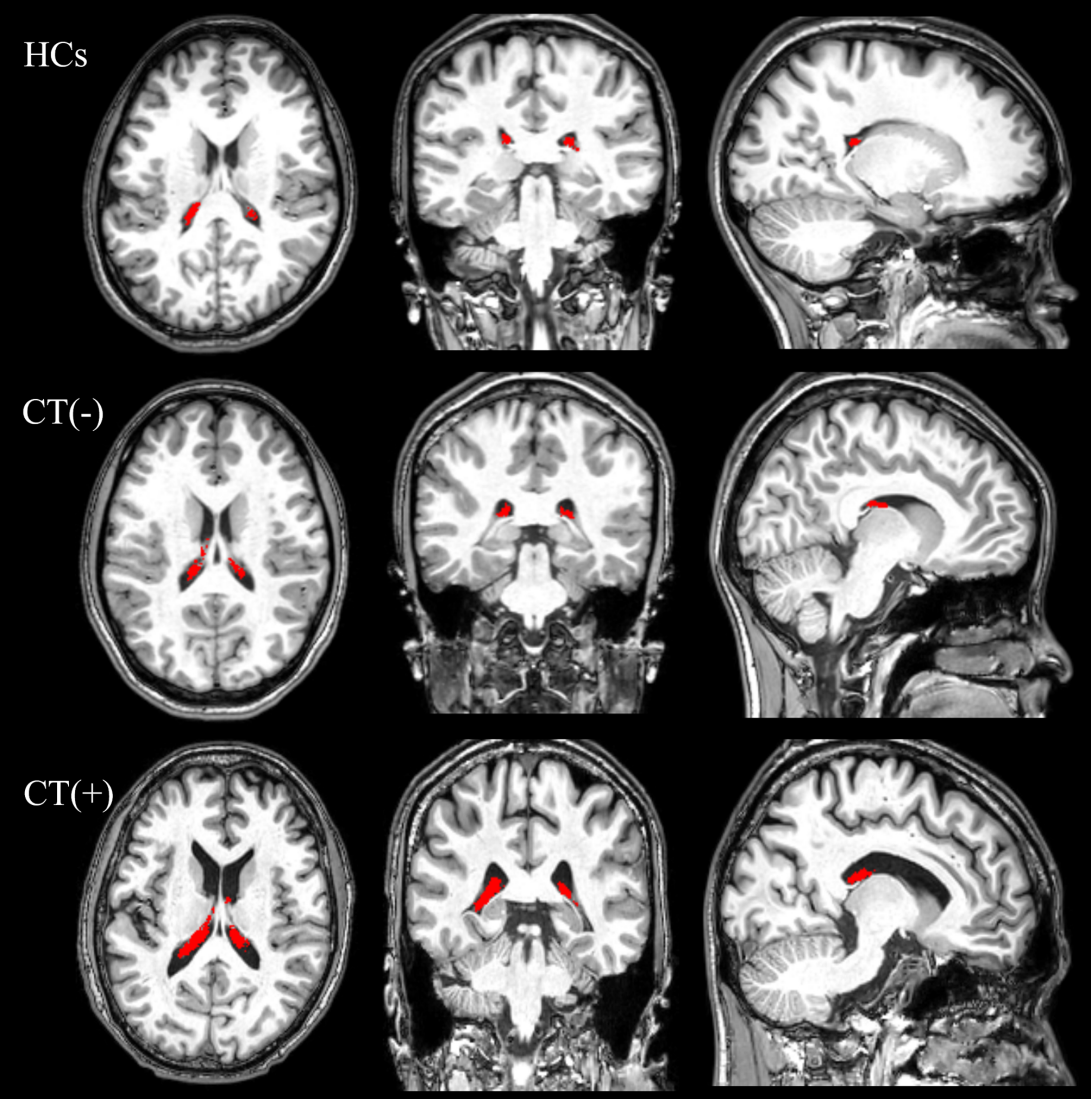
A schematic diagram of the CP volume. The red area represents the CP volume. The images show that the CP volume in the CT(+) group is significantly larger than that in the CT(-) group and the HCs group. CP: Choroid Plexus. HCs: Healthy Control Group; CT(-): Non-Chemotherapy Group; CT(+): Chemotherapy Group.

**Fig. (5):**
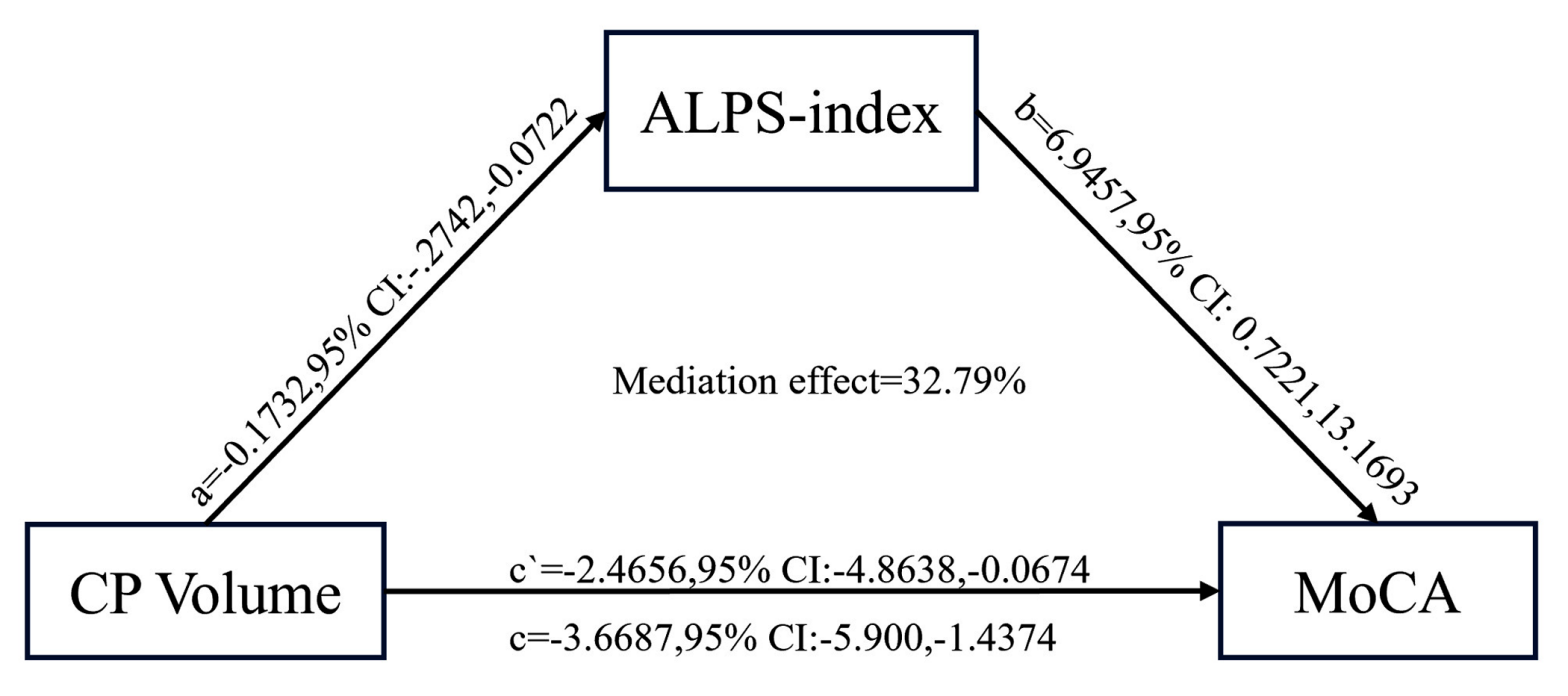
Partial mediation effect of DTI-ALPS index between CP volume and MoCA scores in CT(+) group (indirect effect standardized β=-0.1425, *p <* 0.05, mediated effect=32.79%). DTI-ALPS: Diffusion Tensor Imaging Along the Perivascular Space. CP volume: Choroid Plexus (CP) Volume. MoCA: Montreal Cognitive Assessment.

**Table 1:** Demographic and clinical characteristics.

-	**CT(-)(N=53)**	**CT(+)(N=49)**	**HCs(N=56)**	**F or χ^2^ Value**	***P*-value**
Age (years)	61.79±8.88	62.64±8.64	61.39±8.76	0.287	0.830^a^
Gender (male/female)	26/27	22/27	29/27	0.373	0.783^b^
Education (years)	11.15±1.81	11.20±2.35	11.48±2.09	0.396	0.674 ^a^
Histological types (SqCC/ ACC)	25/28	22/27	-		-
Chemotherapy (cycles)	-	4-6	-		
Chemotherapy regimens (cisplatin/ carboplatin)	-	29/20	-		
MMSE	27.60±1.13	27.31±1.22	27.85±1.49	1.721	0.182 ^a^
MoCA	23.96±2.83	22.41±3.24	26.64±1.37	36.769	<0.001*^,a^

**Note:** Data are represented as Mean ± SD, a: The p values are obtained by using one-way analysis of variance.b: The p-values are obtained by using the χ2 test. *: p<0.05. CT(-): Non-Chemotherapy Group; CT(+): Chemotherapy Group; HCs: Healthy Control Group. SqCC: Squamous Cell Carcinoma; ACC: Adenocarcinoma; MMSE: Mini-Mental State Exam; MoCA: Montreal Cognitive Assessment.

**Table 2:** Comparisons of the DTI-ALPS index and choroid plexus volume among three groups.

-	**CT(-)**	**CT(+)**	**HCs**	**F Value**	***P*-value**
DTI-ALPS index	1.627±0.1798	1.519±0.124	1.621±0.1819	6.729	0.002*
CP Volume	1.545±0.317	1.695±0.0393	1.489±0.418	4.049	0.019*

**Note:** Data are represented as Mean ± SD. *p < 0.05. CT(-): Non-Chemotherapy Group; CT(+): Chemotherapy Group; HCs: Healthy Control Group. ALPS: Analysis Along the Perivascular Space. CP Volume: Choroid Plexus Volume.

## Data Availability

The data that support the findings of this study are available from the corresponding authors [F.Y] and [X.Y] on special request.

## LIST OF ABBREVIATIONS

CRCI: Chemotherapy-Related Cognitive Impairment
NSCLC: Non-Small Cell Lung Cancer
CNS: Central Nervous System
CP: Choroid Plexus
MRI: Magnetic Resonance Imaging
FLAIR: Fluid-Attenuated Inversion Recovery
DTI: Diffusion Tensor Images
EPI: Echo Planar Imaging

## STANDARDS OF REPORTING

STROBE guidelines were followed.
